# Whole Exome Sequencing in a Population With Severe Congenital Anomalies of Kidney and Urinary Tract

**DOI:** 10.3389/fped.2022.898773

**Published:** 2022-08-04

**Authors:** Meredith Harris, Meredith P. Schuh, David McKinney, Kenneth Kaufman, Elif Erkan

**Affiliations:** ^1^Division of Nephrology and Hypertension, Cincinnati Children’s Hospital Medical Center, Cincinnati, OH, United States; ^2^Division of Nephrology, Ann & Robert H. Lurie Children’s Hospital of Chicago, Chicago, IL, United States; ^3^University of Cincinnati College of Medicine, Cincinnati, OH, United States; ^4^Center for Autoimmune Genomics and Etiology, Cincinnati Children’s Hospital Medical Center, Cincinnati, OH, United States

**Keywords:** CAKUT, genetics, amnioinfusion, whole exome sequencing, bladder outlet obstruction

## Abstract

Fetal and neonatal interventions (e.g., amnioinfusions, amniotic shunting, and infant dialysis) have increased survival of infants with severe Congenital Anomalies of the Kidney and Urinary Tract (CAKUT), however, outcomes vary dramatically. Our aim was to perform Whole Exome Sequencing (WES) in a unique severe CAKUT population with the goal to identify new variants that will enhance prediction of postnatal outcomes. We performed trio WES on five infants with severe CAKUT (undergoing fetal interventions and/or those who initiated renal replacement therapy (RRT) within 1 month of life) and their parents as well as three singletons. We identified three potential candidate gene variants (*NSUN7, MTMR3, CEP162*) and validated two variants in known CAKUT genes (*GATA3 and FRAS1*) showing strong enrichment in this severe phenotype population. Based on our small pilot study of a unique severe CAKUT population, WES appears to be a potential tool to help predict the course of infants with severe CAKUT prenatally.

## Introduction

Congenital Anomalies of the Kidney and Urinary Tract (CAKUT) are the leading cause of End Stage Kidney Disease (ESKD) in the pediatric population, representing 30–60% of chronic kidney disease (1, 2). Clinical presentation of these patients varies greatly from asymptomatic patients with normal renal function to ESKD at birth (3). With improved prenatal ultrasound testing, about two-thirds of CAKUT is diagnosed prenatally, with about 50% detected between 18 and 22 weeks of gestation (4).

**FIGURE 1:**
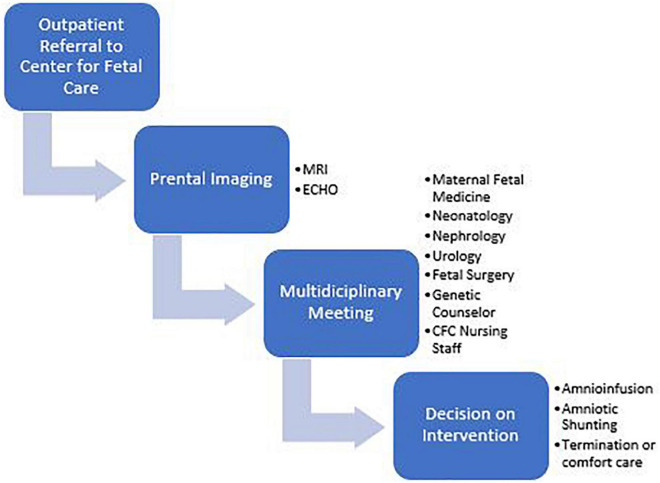
Process diagram for families referred to the center for fetal Care.

**FIGURE 2:**
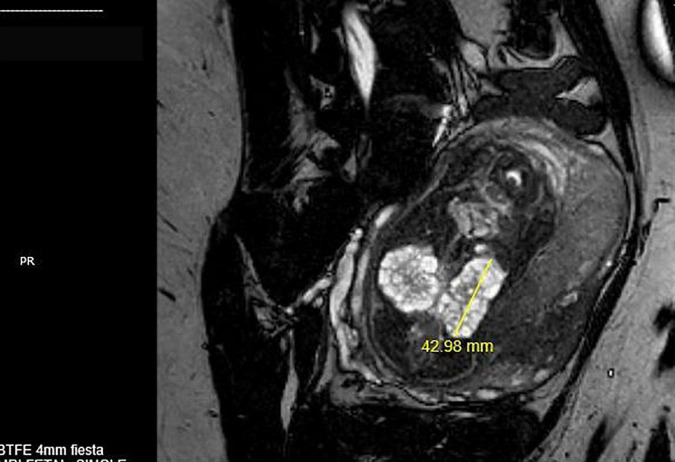
MRI of a singleton fetus in utero at 25 weeks of gestation with bilateral multicystic dysplastic kidneys and absence of amniotic fluid.

Infants with the most severe presentations have oligohydramnios or anhydramnios during gestation. Etiologies include primary structural abnormalities, renal agenesis, or bladder outlet obstruction (BOO). The Center for Fetal Care (CFC) at Cincinnati Children’s Hospital Medical Center is currently one of the few centers in the United States offering amnioinfusions and amniotic shunting for pregnancies with oligo/anhydramnios. Fetal MRI and ultrasound are performed to diagnose the etiology of low amniotic fluid and to aid in parental guidance during a multidisciplinary meeting including neonatology, nephrology, urology, maternal fetal medicine, fetal surgery, palliative care, and neonatology (Figures 1, 2). After this meeting, parents may elect to terminate the pregnancy, not undergo any invasive interventions, or undergo fetal interventions. During amnioinfusions, fluid (Lactated Ringers at our institute) is administered into the uterus at serial intervals. During amniotic shunting, a shunt is placed between the bladder and the uterus to overcome the obstruction in patients with BOO. Both interventions increase fluid availability to the lungs to enhance pulmonary development during the canalicular stage (5). In total, 108 mothers have undergone fetal interventions from 2014 to 2018 in our center, increasing survival to discharge of these CAKUT infants from 17 to ∼50%.

While fetal interventions improve pulmonary survivorship, they do not improve renal outcomes. Our CFC is one of very few centers that offers dialysis for infants as small as 1.8 kg. As a bridge to peritoneal dialysis, we perform prolonged intermittent renal replacement therapy (PIRRT) using the Aquadex^®^ Smart Flow Ultrafiltration Device (6). As a result of successes with fetal interventions and infant dialysis, our center has a unique surviving CAKUT population with a severe phenotype that previously would be incompatible with life. Namely, we have the largest living population of patients with bilateral Multicystic dysplastic kidney (bMCDK), a condition that was previously fatal before fetal interventions as these patients have no amniotic fluid, and subsequently, poor lung development. To date, there is minimal research in living bMCDK populations.

The long-term implications of this previously lethal anomaly are unknown but are imperative for accurate and informed prenatal counseling. CAKUT is known to cover a wide variety of phenotypes with a complex molecular basis with both environmental and genetic contributors. Traditional genetic approaches, such as targeted investigation of single genes, provided the foundation of knowledge of the genetics of CAKUT, covering 5–10% of CAKUT cases (7–10). Genome-wide genotyping has dramatically expanded the list of genes known to cause the disease. More recently, various genetic testing including Whole Exome Sequencing (WES) has identified more than 40 monogenic genes, attributing to up to 20% of CAKUT diagnoses (11, 12). Even with these advances, genetic etiology of the disease is unknown in most cases. Previous work supports that the detection rate of genetic etiologies is higher for bilateral renal anomalies (13, 14).

With a new emerging severe CAKUT population, there is a critical need to provide targeted prenatal counseling *via* identification of genotype-phenotype correlations associated with severe CAKUT prior to aggressive fetal interventions. Based on previous studies, we hypothesize that our phenotypically severe CAKUT population will contain higher numbers of variants in novel kidney disease-causing genes. Here, we establish the framework for WES testing in severe CAKUT during the early postnatal period. As outcomes in our severe CAKUT population remain variable from intrauterine demise to successful kidney transplantation, our goal is to develop new metrics based on genetic testing that will enhance the ability to predict the postnatal outcomes of these infants.

## Materials and Methods

Inclusion criteria included subjects whose mothers underwent fetal interventions (amnioinfusions or amniotic shunting) for severe CAKUT or who started dialysis within 1 month of life. The phenotype of these patients included bMCDK as well as BOO severe enough to warrant fetal interventions for oligo/anhydramnios. Biological parents were included for the purpose of trio WES testing. Infants with minor or moderate CAKUT not requiring fetal interventions as well as infants with bilateral renal agenesis (BRA) were excluded from this study. BRA was excluded as our center does not currently offer intervention to this population (15). All study methods were IRB approved at our center and the delivery hospital centers.

### Sample Collection

For infants, 1 ml of whole blood was obtained for WES. Whole blood samples were collected in an EDTA microtainer, mixed on a nutator, and centrifuged for 5 min at 3 665 G at 4°C. Parental blood (gold standard) or saliva was collected and stored at the internal biorepository to use for WES according to the above methods.

DNA was extracted from whole blood samples using an AutoGen Flex STAR machine *via* precipitation using Qiagen chemistry. A positive and negative control were used to validate the run. Extracted DNA was placed into a cryovial (0.175 ml). This vial was rotated overnight and placed in an incubator for 30 min to ensure even distribution of DNA in the sample. The sample then underwent DNA quantification and a gel quality check. The sample was then stored at −20°C.

### Identification of Genetic Variants by Genetic Analyses

For WES analysis, sequencing was performed on genomic DNA libraries created by fragmentation and annealing of linkers according to the Agilent TruSeq (Illumina) protocol. Exome capture was performed with IDTERPv1 capture kits. Sequencing was performed on the Illumina NovaSeq platforms. Fastq files were aligned to the NCBI human reference genome (build 37) using the Burrows-Wheeler Aligner. Multi-sample variant call format (vcf) files were generated using the Broad Institutes GATK software following their best practices. Variants were filtered based on a 1% minor allele frequency in the general population using both public and internal data sets. Variants underwent quality control filtering to remove sequencing artifacts, were annotated to identify amino acid altering variants, were scored using multiple functional prediction algorithms and were fit to homozygous recessive, compound heterozygous, and *de Novo* genetic models of inheritance. We required the read depth to be >15, genotype quality score >20, and used zygosity based filtering based on the ratio of alternate alleles to reference alleles. Reference alleles required the following: Homozygous reference alleles-ratio less than 0.15, homozygous alternate alleles-ratio greater than 0.85 and heterozygous alleles-ratio between 0.3 and 0.7. In our experience these filters remove ∼99% of the sequencing artifacts.

Variants were prioritized using a multifaceted approach using the GeneCards, NCBI and OMIM databases to assess gene function, gene expression, gene enrichment, and severity of predicted biological impact on gene function using SIFT (16), Polyphen (17), Mutation Taster (18), Mutation Assessor (19), and FATHMM (20). ToppFun was used to further detect functional enrichment of candidate genes (21). We used Franklin by Genoox (22) to classify variants according to the 2015 American College of Genetics and Genomics (ACMG) criteria. These classifications are based on population data, computational and predictive data, functional data, segregation data, *de novo* data, allelic data as well as other additional data (23).

## Results

### Patient Demographics

**FIGURE 3:**
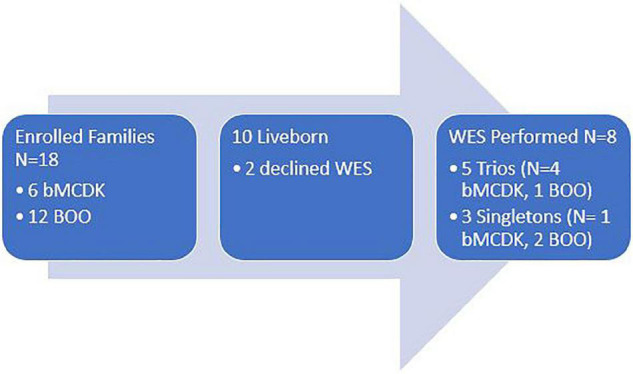
Study enrollment population based on diagnosis and study performed. WES, whole exome sequencing. bMCDK, bilateral multicystic dysplastic kidney. BOO, bladder outlet obstruction.

We enrolled 18 patients along with their parents (Figure 3). From the enrolled patients, we obtained blood from eight liveborn patients for WES. Six patients underwent fetal demise, and 2 patients did not consent for WES. Demographics of this population are in Table 1. Six of these families have the rare diagnosis of bMCDK.

**TABLE 1:** Demographics of the patient population with severe congenital anomalies of the kidney and urinary tract (CAKUT) by diagnosis.

Demographics of patients with severe CAKUT (*N* = 18)	*N* (%)
**Diagnosis**	
bMCDK	6 (33)
BOO	12 (67)
**Gender (male)**	13 (72)
bMCDK	1 (16)
BOO	12 (100)
**Anuric**	12 (67)
bMCDK	6 (33)
BOO	6 (33)
**Death in Utero**	6 (33)
bMCDK	1 (16)
BOO	5 (42)
**Transplant Recipient**	6 (33)
bMCDK	4 (22)
BOO	2 (11)

*bMCDK, bilateral multicystic dysplastic kidney. BOO, bladder outlet obstruction.*

### Whole Exome Sequencing

We performed variant calling on 5 trios (4 bMCDK) and 3 singleton samples (1 bMCDK). We identified 3 novel gene candidates (*NSUN7, MTMR3, CEP162*) and validated known variants of 2 CAKUT genes *GATA3 and FRAS1*. The bMCDK families are designated by F1, F2, F3, and F4. In one bMCDK family, we did not detect any candidate genes. The three candidate gene variants are described in Table 2. In bMCDK family F2, we identified a *de novo* frameshift deletion of five base pairs of *NSUN7* near the terminal end of chromosome 4, a protein coding gene involved in the RNA methylation transferase pathway. *NSUN7* is moderately expressed in the kidney (∼4 RPKM). In bMCDK family F3, we identified a *de novo* SNV of *MTMR3* on chromosome 22 which 3/5 analyses predict to be damaging (C to T exchange). *MTMR3* mRNA is expressed in the renal cortex and medulla at a level of ∼5 RPKM. Additionally, two bMCDK patients shared a non-synonymous SNV in *CEP162* (F1, F2-A to G exchange). This mutation, found in 0.0009% of the population, is predicted to be damaging in 4/5 analyses. All 3 novel gene candidates were classified as variants of unknown significance (VUS) using ACMG guidelines (22).

**TABLE 2:** Three candidate genes and two previously described congenital anomalies of the kidney and urinary tract genes in the bilateral Multicystic dysplastic kidney disease (MCKD) population.

Gene	Chromosome: Variant	Inheritance/Zygosity	ACMG class	bMCDK family	Variant frequency	Predicted tolerance***	Pathway	Gene function	Syndromes affiliated with gene
**Genetic variants of unknown significance in bilateral MCDK families**									
*NSUN7*	4: *De novo* frameshift c.2033_2037delGTTGG	DN	PVS1 PM2	F2	N/a	N/a	tRNA methylation transferase	Protein helix formation	Male infertility, familial restrictive cardiomyopathy
*MTMR3*	22: C to T SNV c.3062C > T	DN	PM2	F3	0.000004%	2/5	Protein Phosphatase, mTOR	Endosomal Trafficking, Autophagy, Cilia Signaling, Apoptosis	Lupus Nephritis, IgA nephropathy
*CEP162*	6: A to G SNV c.209T > C	Enriched het	PM2 BS2	F1, F2	0.0009%	1/5	Axonemal Microtubule Binding	Ciliogenesis, Organelle Biogenesis	Seckel Syndrome, Orofacial Digital Syndrome
*FRAS1*	4: G to A SNV c.9806G > A	DN/het	BP6	F1, F2	0.0037%	1/5	Integrin	Organo-genesis	Fraser Syndrome
*GATA3*	10: C to T SNV c.826C > T	Enriched het	PM2 PP3 PM1	F1	N/A	0/5	T cell transcription factor	Immune response through T cell mediation	Renal dysplasia, sensorineural deafness, hypoparathyroidism

All three variants are extremely rare in the general population according to the Gnomad Database with the deletion of NSUN7 never having previously been described. This table also displays the function of the genes that have been isolated with variants in our bilateral Multicystic dysplastic kidney disease population (MCDK). Through their individual pathways and interaction with nephron development or function, these genetic variants are candidates for patient phenotype. Variants of Unknown Significance are classified according to the 2015 American College of Genetics and Genomics (ACMG) criteria. SNV, single nucleotide variant; bMCDK, bilateral multicystic dysplastic kidney; DN, de novo; Het, heterozygous; PVS1, pathogenic, very strong; PM2, pathogenic, moderate; BS2, benign, strong; BP6, reputable source; PP3, computational data; PM1, pathogenic, moderate; F, family number. ***The variants were analyzed via SIFT, Polyphen, Mutation Taster, Mutation Assessor, FATHMM for tolerance. ACMG, American College of Medical Genetics and Genomics; VUS, variant of unknown significance.

**FIGURE 4:**
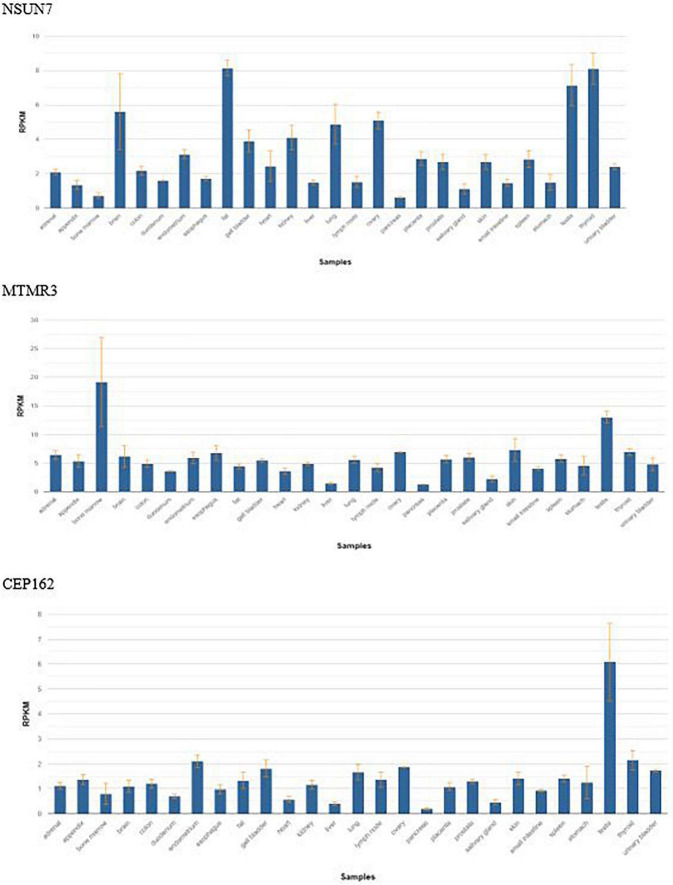
Frequency of gene expression by tissue of the three candidate genes found in bilateral Multicystic dysplastic kidney disease. RPKM, reads per kilobase of transcript.

In 2 bMCDK families, we identified a rare variant in *FRAS1* not associated with Fraser Syndrome. *FRAS1* RNA is expressed extensively in the kidney at a level of ∼4 Reads per Kilobase of Transcript (RPMK) (Figure 4) (24). We identified a rare SNV in *FRAS1* on chromosome 4 in 2 individual bMCDK families (F1 and F2-G to A exchange, present in 0.0037% of the population). This mutation is predicted to be damaging in 4/5 analyses (SIFT, Polyphen, Mutation Taster, Mutation Assessor, FATHMM). Additionally, MCDK family F1 had a *de novo* SNV in *GATA3*, which is a known variant for CAKUT(C to T exchange) (11).

In addition to these 5 genetic variants, we also found 6 genes with rare variants enriched in the GO annotation GO:0001701:*in utero* embryonic development of 526 genes (*P* = 1.3 × 10^–4^ TopGene). These genes include *GATA3* (as previously mentioned), *TTN, TGBR1, CDKN1C, HSD17B2, NCAPG2*.

## Discussion

Previously incompatible with life, infants with severe CAKUT may develop unknown manifestations of their underlying genetic variants. In our pilot study of 18 patients in a unique severe CAKUT population requiring fetal interventions and/or early infant dialysis, we have discovered three novel CAKUT candidate genes and validated two previously established CAKUT genes in our study population as well as identified 6 rare variants implicated in embryonic development. As one of the few centers in North America that performs fetal interventions on infants with severe CAKUT as well as performs infant dialysis *via* PIRRT, we have a unique population of severely affected patients that has not previously been studied. Targeted prenatal counseling and postnatal care is imperative for this growing population.

Little is known about the genetic etiology or clinical outcomes of bMCDK given its high rate of fetal demise without intervention. Ishiwa et al. found that patients with a unilateral MCDK with a contralateral hypoplastic kidney (less severe phenotype than bMCDK) have an increased likelihood of genetic mutations (25). Previous studies also support that the detection rate of a genetic etiology for CAKUT is higher for bilateral renal anomalies (12, 13). This is reinforced in our study, where despite a small patient population, we identified strong genetic variant enrichment. Additionally, identifying variants in *FRAS1* and *GATA3* establishes feasibility of our project through identification of known genes associated with CAKUT, while *MTMR3, CEP162*, and *NSUN7* represent novel gene unique to this severe patient phenotype. The clinical implications of these variants are shown in Table 2 and are further discussed below.

*GATA3* known to be implicated in CAKUT, is a critical regulator of ureteric bud positioning upstream of RET signaling (26). *GATA3* mutations are known to cause HDR syndrome, characterized by hypoparathyroidism, deafness and renal dysplasia (27, 28). The proband in this study did not have hearing loss or hypoparathyroidism (although this is likely masked by the bone mineral disease of ESKD). Importantly, validation of *GATA3* in one bMCDK family validates our study design and supports strong enrichment in a small sample size.

In our study, we identified several rare variants in *FRAS1* not associated with Fraser Syndrome. Of note, one rare variant was present in 2 different bMCDK families, further pointing to enrichment. *FRAS1* encodes the protein FRAS1, an extracellular matrix protein. Mutations in this gene can be associated with Fraser syndrome, an autosomal recessive mutation associated with cryptophthalmos, cutaneous syndactyly, and renal agenesis (29–31), as well as isolated CAKUT (12, 32, 33). Saisawat et al. described four heterozygous truncating mutations in *FRAS1* as a potential cause of non-syndromic CAKUT in a pooled exome analysis of 40 CAKUT patients (32). Notably, the phenotype in this study was less severe than ours with 29 patients having unilateral MCDK only. However, one patient in this pooled exome study with a compound heterozygous mutation in *FRAS1* had a sibling with previous intrauterine demise secondary to bilateral renal agenesis, suggesting various *FRAS1* mutations may cause more severe renal phenotypes. This is further supported by a recent publication identifying biallelic *FRAS1* variants in four cases of bilateral renal agenesis (34).

This study introduced three candidate genes implicated in severe CAKUT. In one bMCDK family, we detected a *de novo* frameshift deletion in *NSUN7. NSUN7* is part of a 6 gene family of the NOL1/NOP2/SUN domain that catalyzes the methylation of cytosine to 5-methycytosine (m^5^c) (35, 36). *NSUN7* encodes a methyltransferase protein by the same name and has been implicated in familial restrictive cardiomyopathy as well as male infertility through the mechanism of sperm dysmotility (37). *NSUN7* encodes for an RNA methyltransferase and is currently not described in renal development literature or associated with the CAKUT phenotype. However, single-cell RNA seq expression data of the mid-gestation human kidney demonstrates *NSUN7* sharing expression patterns of known progenitor markers, including *CITED*1 and *EYA1* (38). *NSUN7* is also expressed in the distal portion of the developing human (39) and murine kidney (40). *NSUN2-NSUN7* has been implicated in aberrant murine embryogenesis in multiple organ systems including the brain and liver, often through inappropriate protein helix formation and decreased expression of genes that require methylation by *NSUN7* (36, 41). In studies on *NSUN7* mutations in asthenospermic men, a single SNV lead to a change in the structure of the helix, coil and strand of the protein with impairment of protein function (41). Inappropriately folded proteins induce the Unfolded Protein Response which triggers a cytotoxic pathway, leading to apoptosis and impairment in cell function and structure (42). Additionally, *NOP2/NSUN1* and *NSUN2* are implicated in epigenetic modifications to create transcriptionally active chromatin (43). Due to the role of *NSUN7* in epigenetic methylation of proteins, depletion of NSUN leads to diminished expression of the enhancer RNA of these proteins, especially ones that rely on cytosine modification (36). Unfortunately, studies on the role of the NOL1/NOP2 SUN domain in renal embryogenesis are lacking (35). However, impaired cystine methylation during renal development has been implicated in renal pathologies including the *SIX2*, *PAX2* gene and the TGF-β pathway leading to podocyte loss, fibrosis, mesangial proliferation, and tubular dysgenesis (44–47). Further research in the CAKUT patient population will be critical to understand how *NSUN7* is involved in kidney development.

Additionally, we identified a rare SNV in *MTMR3* in one bMCDK family. *MTMR3* belongs to the protein-phosphatase family and is structurally similar to myotubularin. It is highly expressed in the ureteric lineage of the developing mouse kidney (40) and the proximal s-shaped body of the human nephron (39). The myotubularin family regulates endosomal trafficking, apoptosis, and autophagy. *MTMR3* inhibits the activity of mTORC1, regulating autophagy (48–51). *Mtor* deletion in mice have severe paucity of glomeruli which lead to death after birth as *Mtor* regulates cell growth, proliferation and protein synthesis (52). Variants of *MTMR3* have also recently been implicated in lupus nephritis and IgA nephropathy, implicating *MTMR3* as important for renal function and immune regulation (53).

Similar to *MTMR3, CEP162* is also moderately expressed throughout the renal tubules (54). In the developing kidney, *CEP162* is highly expressed from nephron progenitor to differentiated structures (55), implicating its role early in development. The protein *CEP162* is an axoneme-associated protein that promotes transition zone assembly of the cilia (56). Loss of *CEP162* arrests ciliogenesis at the transition zone of the cilia in animal models (56). Dysfunctional cilia, leading to “ciliopathies” have been linked to more than 180 proteins (57–59). Primary cilia are located on the polarized epithelial cells of the renal tubule and are involved in sensation rather than motility (60). Dysregulated cilia-dependent processes have been attributed to cyst formation and progression of chronic kidney disease (61). MCDK has not previously been considered a ciliopathy, but the strong enrichment of these genes supports an early developmental spectrum of ciliopathy presentation resulting in a severe phenotype.

Limitations of this study include a small sample size. However, this pilot data supports strong enrichment of candidate genes in a novel severe phenotype population. This study is limited by exclusion of patients with BRA. In a study by Riddle et al. completed at our institution, all 8 patients who underwent serial amnioinfusions for BRA died either *in utero* (*n* = 6) or within 30 days of delivery (*n* = 2) (15). As a result of this study, fetal interventions and infant dialysis are no longer offered to mothers of infants with BRA at our institution. It is promising that with even the small number of samples sequenced, we are identifying new variants in CAKUT genes as well as suggestions of gene enrichment in pathways involved in CAKUT.

Even with these limitations, this study lays the groundwork for identifying additional genetic causes for severe CAKUT. The goal of this study is to provide individualized and accurate prenatal and postnatal counseling for families with anomalies previously incompatible with life. Clinically, there is a wide range of variability in outcomes in this population including survival to delivery, pulmonary survivorship, degree of respiratory support, need for urological interventions, length of hospitalization, survival to hospital discharge and degree of developmental delay, which have been difficult to predict. We aim to enhance our genetic data with biomarker data currently being investigated to develop a polygenic risk score (PRS) to predict and screen for disease severity early in pregnancy, as well as counsel families on other associated findings with these genetic mutations. Polygenic risk scores have been used successfully in other conditions such as diabetes mellitus and urinary tract stones (62, 63). In our model, weight will be placed differently based on (1) known CAKUT risk variants, (2) variants in known CAKUT genes of unknown significance, and (3) variants in plausible genes.

Based on our small pilot study of a unique severe CAKUT population, WES testing appears to be a promising diagnostic tool to help predict the course of infants with severe CAKUT prenatally. We believe this data, together with clinical outcomes, will improve our ability to counsel parents and improve clinical outcomes as well as help us further understand the role of the intricate genetic network in renal embryogenesis.

## Data Availability Statement

The datasets presented in this study are not readily available because it may be possible for this specific de-identified genetic data to be re-identified. Requests to access the datasets should be directed to MH.

## Ethics Statement

The studies involving human participants were reviewed and approved by the Cincinnati Children’s Hospital IRB. Written informed consent to participate in this study was provided by the participants’ legal guardian/next of kin.

## Author Contributions

MH and EE: study conception and design. MH, EE, and DM: data collection. MH, MS, KK, and EE: analysis and interpretation of results and draft manuscript preparation. All authors reviewed the results and approved the final version of the manuscript.

## Conflict of Interest

The authors declare that the research was conducted in the absence of any commercial or financial relationships that could be construed as a potential conflict of interest.

## Publisher’s Note

All claims expressed in this article are solely those of the authors and do not necessarily represent those of their affiliated organizations, or those of the publisher, the editors and the reviewers. Any product that may be evaluated in this article, or claim that may be made by its manufacturer, is not guaranteed or endorsed by the publisher.
